# Effectiveness of cognitive behavioral therapy with yoga in reducing job stress among university lecturers

**DOI:** 10.3389/fpsyg.2022.950969

**Published:** 2023-01-05

**Authors:** Ntasiobi C. N. Igu, Francisca N. Ogba, Uchenna N. Eze, Michael O. Binuomote, Chinyere O. Elom, Emeka Nwinyinya, Joy I. Ugwu, David O. Ekeh

**Affiliations:** ^1^Department of Educational Administration, Alex Ekwueme Federal University Ndufu-Alike, Abakaliki, Nigeria; ^2^Department of Educational Foundations, Faculty of Education, University of Nigeria Nsukka, Nsukka, Nigeria; ^3^Department of Technical and Vocational Education, Alex Ekwueme Federal University Ndufu-Alike, Abakaliki, Nigeria; ^4^Department of Psychology, Faculty of Social Sciences, University of Nigeria, Nsukka, Nigeria

**Keywords:** cognitive behavioral therapy, yoga, job stress, university lecturers, teachers, health, wellbeing, randomized control trial

## Abstract

**Introduction:**

Job stress is highly prevalent in the workforce worldwide, and tends to threaten employees’ physical and mental wellbeing, reducing organizational outcomes. The negative impacts of workplace stress on academics have been found to disproportionately interfere with both institutional research productivity and students’ learning outcomes. This study analyzed data from a randomized control trial, to validate the effectiveness of cognitive behavioral therapy combined with yoga in treating job-related stress among lecturers from two Universities in South-East, Nigeria.

**Methods:**

Participants included 93 academic staff members from two Federal Universities in Enugu and Ebonyi States in Nigeria. We assigned participants to Y-CBT (*N* = 46) and waitlist control (*N* = 47) groups using random sampling techniques. A 2-h Y-CBT program was delivered weekly for a period of 12 weeks. Two instruments were used to collect data for the study. Single Item Stress Questionnaire (SISQ) was employed to identify the potential participants, while the teachers’ Stress Inventory (TSI) was served for data collection at baseline, post-intervention, and follow-up tests. Mean, standard deviations, *t*-test, statistics, and repeated measures Analysis of Variance, were used to analyze data for the study.

**Results:**

Results revealed that the perception of stressors and stress symptoms reduced significantly at post-test and follow-up assessments following Y-CBT intervention.

**Discussion and conclusion:**

The outcomes of this study support the prior that Y-CBT is valuable for harmonizing mind and body for a stable psychological state. The conclusion was that Y-CBT can minimize the perception of stressors and stress manifestation among university lecturers.

## Introduction

Stress involves physical, mental, and/or emotional reaction to demand-resource imbalances that cause bodily tension that negatively affects people (American psychological Association, 2012; Okeke and Dlamini, 2013). The causes of stress across the world are mostly related to work conditions and individual personal situations (Azeem and Nazir, 2008; Nwokeoma et al., 2019). High job stress levels have been found among majority of lecturers who are teachers in Universities (Li and Kou, 2018; Meng and Wang, 2018). According to consensus, the teaching profession is one of the most stressful occupations all over the globe (Kyriacou, 2001; Busari, 2018). Teachers are on the top list of employees most affected by stress when compared with other professionals (Lever et al., 2017; Ogba et al., 2019).

University teachers particularly present a higher level of stress across cultures and settings (Blix et al., 1994; Slišković and Seršić, 2011; Masuku and Muchemwa, 2015: Li and Kou, 2018; Meng and Wang, 2018). Ogbuanya et al. (2017) affirmed that the teaching staff of the universities is the most stressed workers and over 60–70% are reported with mental health disorders associated with job stress due to the enormity of the workload. Studies tend to show that lecturers’ stress emanate mainly from school administration and management, time pressure, and workload, coping with changes, role conflict, maintaining discipline, curriculum ambiguity, dealing with colleagues, and self-esteem (Winter, 2009; Arokium, 2010; Gold and Roth, 2013). More critical challenges are those associated with teaching, scientific research and personal development pressures (Masuku and Muchemwa, 2015; Li and Kou, 2018; Meng and Wang, 2018).

University lecturers in Nigeria encounter disproportionate demands to meet up with the academic, social, and emotional demands of their students (Ekundayo and Kolawole, 2013; Okeke and Dlamini, 2013). Stress among university lecturers gets worsened by an emotionally taxing and potentially frustrating promotion syndrome of research and publication titled “publish or perish” (Archibong et al., 2010; Akinmayowa and Kadiri, 2014), which now has been hardened by a strict specification for publication in Thomson Reuters’ journals in some Universities in Nigeria. Other challenges include strikes and academic calendar disruptions (Ogba and Igu, 2012), delays and irregular payment of salary (Ofoegbu and Nwadiani, 2006), poor working conditions (Okeke, 2013), lecturers’ work lives (Archibong et al., 2010), and poor facilities. All these and similar challenges enshroud academics, getting a good number of them to a state of pathological feelings of emotional drainage and stress.

Stress is characterized by unpleasant negative emotions and physical exhaustion that cause worry, pain, anxiety, depression, frustrations, and burnout (McCarthy et al., 2009; Ali et al., 2013). According to the World Health Organization [WHO] (2016), stress accounts for health problems such as chronic fatigue and muscular pain. Due to its economic and health burdens, as well as a dramatic increase in the incidence worldwide (Ballas, 2009; Wanjiku, 2015; Camacho, 2017), stress is increasingly attracting researchers’ interest. The escalating effects of stress have been found to account for about 20% of referrals to health centers globally (European Commission, 2000; Marten and Wilkerson, 2003; Milutinović et al., 2012). Lecturers who are overwhelmed by workplace stress tend to encounter persistent emotional fatigue, depersonalization, and a sensation of a poor value of achievement. Consequently, a good number of lecturers leave the teaching profession or even get frustrated in a way that negatively affects their health (Ingersoll, 2001; Darling-Hammond, 2010).

Others who remain in the profession in the face of a significant level of emotional exhaustion may stand the risk of imparting negative academic and social-emotional experiences to their students (Camacho, 2017; Harish and JeyaPrabha, 2018). Under this condition, they may be quick to utilize punitive responses and derogative labels on students’ behavior that do not model emotional regulation (Deb et al., 2015; Ogbuanya et al., 2017). All these not only impinge on the productivity and creativity of university teachers but also affect negatively their overall wellbeing and morale, worst still, exposing them to somatic and psychiatric disorders which are inimical to the achievement of educational aims (Hassard et al., 2014). Reduction in stress could help increase performance (Fisher, 2011), support improved social associations (Barmby, 2006), and may reduce sick leave and absenteeism (De Neve et al., 2013).

Hence, stress management interventions are needed to alleviate the effects of stress on both employees and their organizations. Researchers have identified two techniques to address the impact of stress based on their effectiveness and potency. For instance, cognitive behavioral therapy (CBT) (Camacho, 2017), and Yoga (Cramer et al., 2016) have been reported as effective techniques. CBT is one of the psychotherapeutic treatments that have been widely used across contexts for modifying daily behavior. The core tenet of CBT is challenging maladaptive cognitions or irrational beliefs which account for automatic thoughts about a particular situation in a person. CBT aims to positively change the maladaptive cognitions that fan the embers of emotional distresses and improve emotional state (Ali et al., 2013; Orji and Yakubu, 2020). CBT commonly involves finding out the events that account for negative feelings, thoughts, and behaviors, and questioning the assumptions that might be responsible for such outcomes; gradually discarding such negative assumptions to take helpful realities that produce more positive outcomes.

Empirical evidence suggests that CBT is efficacious in handling poor psychological health conditions, including stress, anxiety, and depression (Ogba et al., 2019). CBT is also found efficacious in supporting individuals’ quality of life, especially where there are psychiatric disorders (Jasmine, 2010; Brown and Valenti, 2013). CBT is a planned time-and-cost-effective therapeutic approach that capitalizes on current problems to validate clients’ coping mechanisms (Dobson and Dozois, 2010; Ruotsalainen et al., 2015). CBT treatment for university lecturers can assist them to determine the causes of their stress, acquire functional skills for managing stress, and acquaint them with skills for managing other similar situations subsequently.

According to Allen et al. (2018) CBT skills are effective for psychosomatic outcomes, and yoga practices are more effective for increasing positive outcomes. Therefore, given that CBT focuses on shifting outlooks and belief systems that contribute to stress, combining it with yoga techniques could further harmonize mind and body for increased positive outcomes (Kadden, 2001; Forfylow, 2011). Yoga is a complementary therapy that involves physical exercise. Yoga combines the psyche, spirit, and body to uphold the welfare of people, and is used in plummeting psychosomatic issues such as stress (Maddux et al., 2018). It focuses on physical exercise, breathing, and meditation (Granath et al., 2006; Bridges and Sharma, 2017) to assist one to reach bodily and psychological calmness (Math and Srinivasaraju, 2010; Nagendra, 2013). Yoga minimizes an array of psychological challenges by connecting bodily and psychological practices (Chong et al., 2011; Khalsa et al., 2015; Bragard et al., 2017).

According to Eskandar and Sasan (2015), yoga is a very useful technique for decreasing stress-related troubles, including anxiety, depression, burnout, and stress. Additionally, yoga effectiveness has been validated in field studies, strengthening its invaluable place in curing psychosomatic issues, especially in occupational health practices (Rahimi and Bavaqar, 2010; Naveen et al., 2013; Bridges and Sharma, 2017). Furthermore, yoga attacks perceptions that escalate mental health issues like worry and rumination, thereby improving physical health (Ross and Thomas, 2010; Li and Goldsmith, 2012; Ali et al., 2013; Halliwell et al., 2018). Yoga can be effective either as a complementary therapy or as a separate treatment (Chong et al., 2011; Forfylow, 2011; Khalsa et al., 2015; Bragard et al., 2017). This study adopted a complementary approach by combining yoga with CBT in managing stress among university lecturers in trial research.

Growing pieces of evidence affirm the invaluable benefits of complimenting CBT and Yoga (Y-CBT) for elevated health outcomes (Granath et al., 2006; Sharma et al., 2010; Khalsa et al., 2015; Dore, 2016; Allen et al., 2018; Brenes et al., 2018; Grensman et al., 2018). To the best of our knowledge, there is a dearth of empirical studies utilizing Y-CBT for the treatment of stress among lecturers in Nigerian Universities. In this control trial, we extended the research by implementing a Y-CBT to help university lecturers in managing job stress in a federal university in Nigeria. The outcome of this study will be supportive to students, lecturers, as well as the university community and will ultimately improve school outcomes. We, therefore, put forward that the stress level of the participants would reduce significantly after completing the Y-CBT intervention program, and that the minimized stress would be sustained across a 3 months follow-up assessment.

## Materials and methods

### Study design

We used a waitlist control intervention trial designed with pretest, post-test, and follow-up assessments (Wu and Sullivan, 2009; Desveaux et al., 2016; Dike et al., 2021).

### Ethical consideration

We obtained ethical approval from the Faculty of Educational research ethics Committees of a Federal University in Nigeria. Moreover, the research ethical standards specified by the General Assembly of the World Medical Association (2014) and the American Psychological Association (2017) guided the study. All study participants gave written consent to participate in the study as part of the inclusion procedures. This study is also part of a project registered with the AEA RCT trial Registry with an identity number: “AEARCTR-0005532.”

### Measures

#### The single item stress questionnaire (SISQ)

The SISQ was used to ascertain the eligible participants by identifying lecturers with symptoms of stress as one of the inclusion/exclusion criteria. SISQ has shown good psychometric properties in stress research across contexts (Arapovic-Johansson, 2020; Dike et al., 2021; Obiweluozo et al., 2021), with Chrombach reliability indicator between 0.80 and 0.86. The one-item instrument reads: “stress means a situation when a person feels tense, restless, nervous, anxious, or unable to sleep at night because his or her mind is troubled all the time. Do you feel that kind of stress these days?” The SISQ is measured in a 5-point scale ranging from 1-“not at all” to 5-“very much.” In this study, we adopted the score from 3 to 5 (indicating moderate to high-stress level) as the benchmark for inclusion of participants. To further ascertain the context-based reliability of the SISQ, we administered the instrument to 20 university lecturers in Nigeria and found a reliability index of 0.79, showing that the measure is reliable for Nigerian University lecturers.

#### The teachers’ stress inventory (TSI)

Teachers’ stress inventory (Fimian, 1984) is a questionnaire 49-item that are rated on a five-point Likert scale. The TSI assesses stress in ten subscales, covering two major components of stress (Stress Source-SS and Stress Manifestations-SM). Five subscales, including time management, work-related stressors, professional distress, discipline and motivation, and professional investment measures stress sources. Five subscales of emotional manifestations (such as anxiety, depression, etc.), fatigue manifestations (e.g., changes in sleep, exhaustion, etc.), cardiovascular manifestations (blood pressure, heart rate, etc.), gastronomical manifestations (stomach pain, cramps, etc.) and behavioral manifestations (use of prescription drugs/alcohol, sick leave, etc.) measure SM. The TSI has been found to have good psychometric properties in South Africa (Fimian and Fastenau, 1990; Boshoff, 2011). To establish the usability of the instrument among university teachers in Nigeria, the TSI was trial-tested on 63 lecturers. Data collected were subjected to Crombach’s alpha statistics and yielded a good reliability coefficient (α = 0.81).

### Participants and procedures

The participants were 93 university lecturers, made up of males (*n* = 49) and females (*n* = 44) in 2 Federal universities in Nigeria. After the notification and invitation to the research, 125 possible participants volunteered to participate. The potential participants were checked for eligibility based on the inclusion criteria set by the researchers. The inclusion criteria are: (i) the participant must score up to 3–5 in the Single-Item Measure of Stress Symptoms, showing moderate to high stress levels; (ii) absence of a major emotional disorder like anxiety and/or depression; (iii) absence of chronic illnesses, such as diabetes (iv) not taking any pharmacological treatment within the time of the research; (v) willingness to give personal contacts; and (v) participants signed written consent of availability throughout the study period. Some potential participants were excluded from participating in the research due to not meeting the set criteria.

During the sampling process, two of the researchers visited the two universities that participated in the study, in the company, of four trained research assistants. The lecturers were notified of the program *via* their faculty board meetings as well as the dissemination in their faculty whatsApp and bulk SMS. A total of 125 potential volunteers indicated interest to be included in the program.

**FIGURE 1:**
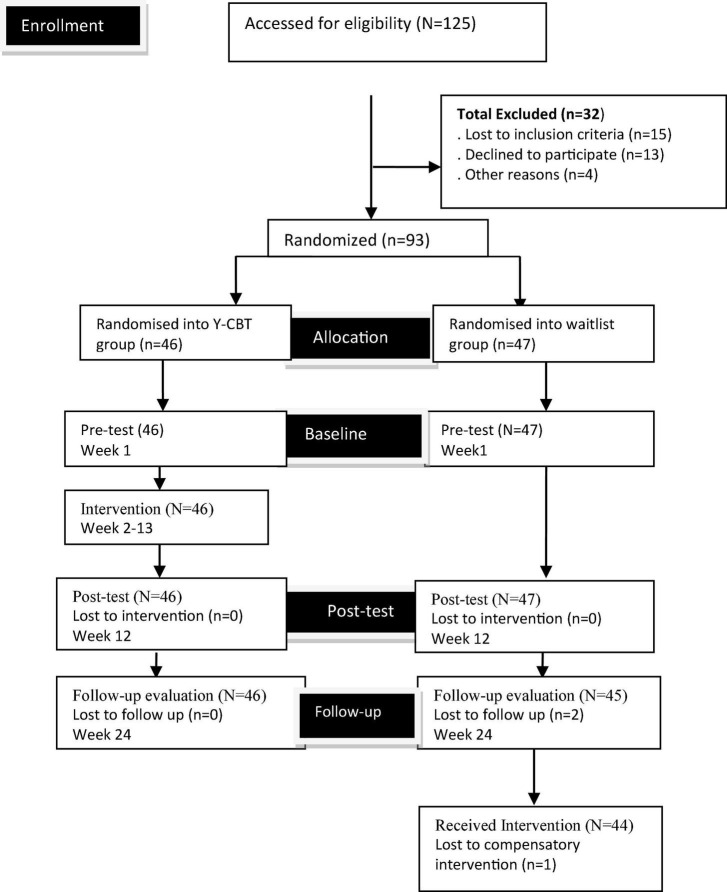
Design/participants’ flow chart.

Out of 125 potential participants who screened for the study, 15 did not meet the inclusion criteria, 13 declined, and 4 persons could not continue for other reasons. Therefore, 93 possible participants who met the inclusion criteria were assigned to Y-CBT group (46 participants) and waitlist control group (47 participants) through a simple random technique (see Figure 1). Sequence allocation software was used for the allocation of the participants into groups (participants were asked to pick 1 envelope containing pressure-sensitive paper labeled with either Y-CBT or WLG-Waitlist Group from a container). We concealed information regarding randomization from the participants until the assignment of the intervention. After the allocation of the groups, we created two separate group WhatsApp platforms for participants in both groups.

Afterward, we collected the baseline data from both the Y-CBT group and the waitlist group (WLG) (Time 1), using TSI. Then, Y-CBT participants received a 2 h weekly Y-CBT intervention for 12 weeks (see intervention sessions). Each of these sessions was proceeded by practice exercise. Two weeks after the 12 weeks intervention, a post-test (time 2) was administered to both Y-CBT and WLG using TSI. Furthermore, follow-up data (Time 3) was collected in a follow-up meeting, held 3 months after the post-test. The same instrument (TSI) was used to collect 3 months follow-up (Time 3) (see Figure 1). Finally, after the 3 months follow-up assessment, the wait-listed group received the compensatory intervention that ran for 12 weeks. This followed the same procedure used for the Y-CBT group.

The Y-CBT intervention sessions were facilitated by two of the researchers who are in collaboration with four research assistants (experts in CBT, Yoga, and occupational therapy) who were Ph.D students. Reminder messages were sent *via* the WhatsApp platform to the participants a day before each meeting, and early morning each day of the intervention meetings.

Table 1 shows the distribution of participants’ demographic information in the Y-CBT and Waitlist Control groups. The mean age of the participants was 44.27 ± 10.0. On the whole, 49 (52.69%) of the participants were males, while 44 (47.31%) were females 25 (26.88%) male and 21 (22.58%) female participants were in the Y-CBT group, while 24 (25.80%) males and 23 (24.73%) females were in the control group. A total of 6 (4.45%) of the participants were single, 55 (59.14%) were married; 11 (11.82%) were divorced, and 22 (58%) had lost their partners. A total of 15 (16.13%) participants were at the rank of assistant lecturer; 29 (31.18%) were lecturers II; 27 (29.03%) were lecturer I; 19 (20.43%) were senior lecturers and 3 (3.22%) were Professors 10 (10.75%) had 1–5 years of experience as lecturers; 32 (34.40%) had 6–10 years of experience; 48 (51.61%) had above 10 years while 3 (3.22%) did not specify.

**TABLE 1:** Participants’ demographic information.

Variable		Y-CBT group (*n* = 46)	Waitlist group (*n* = 47)	Total (*n* = 93)
Age (SD)		43.55 ± 10.0	45.0 ± 50.0	44.27 ± 10.0
**Gender**				
	Male	25 (26.88%)	24 (25.80%)	49 (52.69%)
	Female	21 (22.58%)	23 (24.73%)	44 (47.31%)
**Marital status**				
	Single	4 (4.30%)	2 (2.15%)	6 (4.45%)
	Married	25 (26.88%)	30 (32.25%)	55 (59.14%)
	Divorced	7 (7.52%)	4 (4.30%)	11 (11.82%)
	Widow/er	10 (10.75%)	11 (11.83%)	22 (58%)
**Rank**				
	Assistant lecturer	6 (6.45%)	9 (9.67%)	15 (16.13%)
	Lecturer 2	16 (17.20%)	13 (13.97)	29 (31.18%)
	Lecturer 1	13 (13.97%)	14 (15.05%)	27 (29.03%)
	Senior lecturer	9 (9.67%)	10 (10.75%)	19 (20.43%)
	Professor	2 (2.15%)	1 (1.07%)	3 (3.22%)
**Years of experience**				
	1–5 years	5 (5.37%)	5 (5.37%)	10 (10.75%)
	6–10 years	18 (19.35%)	14 (15.05%)	32 (34.40%)
	Above 10 years	21 (22.58%)	27 (29.03%)	48 (51.61%)
	Unspecified	2 (2.15%)	1 (1.07%)	3 (3.22%)

SD, standard deviation; Y-CBT, yoga and CBT; n, number. Above table shows the demographic distribution of the participants in the experimental and control groups.

### Intervention

In collaboration with two experts (one in CBT and the other in Yoga), we developed a Y-CBT manual to blending CBT strategies with after-session Yoga exercises as used in Dike et al. (2021). The CBT sessions were based on “ABCDE” model (Activating event, Beliefs, Consequences, Disputing, and Effective new philosophy). ABCDE was used to identify, assess, revalidate, and counter dysfunctional beliefs associated with work experiences. The key purposes of Y-CBT were to (1) use ABCDE group therapeutic model (CBT) in “disputing”—countering lecturers’ job-related illogical and dysfunctional viewpoints and to substitute them with helpful and efficient beliefs (DiGiuseppe et al., 2014; Ogbuanya et al., 2017). (2) Use yoga to decrease the emotional manifestations of stress, and help the lecturers out of the vicious circle of negative cognition through victory Meditation, yoga techniques, affirmations, and corporeal exercises (Khalsa et al., 2015). This structure enabled a targeted mind/body approach to stress perceptions, responses, and symptoms.

**FIGURE 2:**
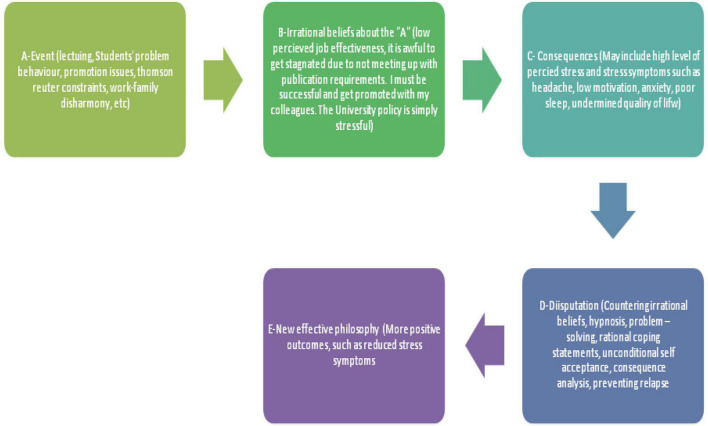
The ABCDE model of lecturers’ stress.

The ABCDE was used to explain the links between activating events (A) in the lecturing jobs and promotion; dysfunctional thoughts, beliefs, or cognitions arising from those events (B); the emotional and behavioral consequences of the beliefs (C) (DiGiuseppe et al., 2014). Activating events (A) in lecturing in the university could include handling students’ behavioral problems, extra workload, publication challenges, and other personal experiences, which may trigger irrational beliefs (B) resulting in negative consequences/effects (C) such as stress. Disputation techniques (D) are used to remove the maladaptive, unhelpful, and self-limiting attitudes and cognitions about job experiences (David and Szentagotai, 2006; DiGiuseppe et al., 2014; David, 2015). It involves countering irrational thoughts with more functional ones (Ellis, 1995). Thus, as participants get conscious of their automatic thoughts, they counter such limiting thoughts and come up with a more effective philosophy (E). The ABCDE modality used in this study were adopted from earlier studies as diagrammatically represented in Figure 2 (see the diagram below).

Complementary yoga strategies used were physical/posture exercises, breathing practices and meditation practices (Butera et al., 2015), delivered after each CBT session. The skills to reduce the absurdity between feelings and perception were taught by linking physical symptoms to cognition and emotion to strengthen physical, mental, and emotional health. Yoga enabled the participants to understand and appreciate the interaction between their bodily sensations and emotional feelings. Specifically, we utilized 10 asana posses, including Tadasana (Mountain pose); Vrikshasana (Tree Pose); Adho Mukha Svanasana (Downward Facing Dog Pose); Trikonasana (Triangle Pose); Kursiasana (Chair Pose); Naukasana (Boat Pose); Bhujangasana (Cobra Pose); Paschimottanasana; Child’s Pose and Sukhasana (Shivangana, 2018).

Breathing exercises were the basic gasp awareness, including Ujjayi Pranayama (Victorious Breath or Ocean Breath); Kapalabhati Pranayama (Breath of Fire or Skull-Shining Breath); Kumbhaka Pranayama (Breath Retention), Nadi Shodhana Pranayama (Alternate-Nostril Breathing). For meditation exercises, we explored getting quiet, calm, and focused; relaxation and mind slowed down, and staying positive through focusing on things that bring peace to the mind and making efforts to shed the unhelpful thoughts that impede health. Through meditation, the participants may turn out to be more watchful of their mind and body harmony.

Thus, in the 12-week Y-CBT module, CBT intervention was complemented by conventional yoga practices, involving information, posing, breathing and meditation exercises, worksheets, images, examples, homework exercises, and a template for progress feedback. We adopted the Y-CBT manual developed and used by Dike et al. (2021). According to the manual, the psychological mechanisms used were familiarization, assessments, goal-setting, problem identification, discussion, disputation, home-work, problem–solving, rational coping statements, unconditional self-acceptance, consequence analysis, cognitive restructuring, guided imagery, relaxation techniques, hypnosis, decision making, physical exercise, physical exercise, medication. The module was presented in three phases, including initial phase, intervention phase, and conclusion/revision phase. The module is summarized below:

#### Phase 1 (initial phase): Week 1–2: Introduction and baseline data collection

The first meeting in this phase (week 1), involved establishing an alliance with the clients; establishing a working atmosphere with the clients; collection of pretest data; setting goals for the intervention. At the second meeting (week 2), the therapists helped the clients to create a problem list of work challenges that cause stress. The therapist introduced the Y-CBT model and explained the complementary role it can play in reducing stress. The yoga exercises used for the study were introduced and explained to the participants as stated in the preceding. Pictures of yoga postures were shared.

#### Phase 2, weeks 3–11 intervention phase

At the first session of the intervention (Week 3), the therapist helped participants to recognize and counter irrational beliefs and worldviews about the job that may constitute stress. This was done by highlighting illogical thinking that follows an unfavorable job experience and encouraging rational beliefs and thoughts. Regarding Yoga, the participants were guided through the ten asana possess—Tadasana, Vrikshasana, Adho Mukha Svanasana, Trikonasana; Kursiasana; Naukasana; Bhujangasana; Paschimottanasana; Child’s Pose and Sukhasana. One hour each was used for CBT and yoga practices.

Furthermore, during sessions 4–6, both the therapist and clients engaged in the disputation of irrational beliefs associated with job experience and replaced them with rational ones using the ABCDE framework. Emphases were laid in developing a rational worldview, rational thoughts, and practices in their jobs. Other activities included linking job stress with irrational beliefs; leading clients to discover how their thinking affects their emotions. Weakening unhelpful thoughts associated with lecturing job. This session is concluded with an hour yoga practices. Breathing exercises were practiced after CBT in this session, and it involved basic gasp awareness, including Ujjayi Pranayama; Kapalabhati Pranayama; Kumbhaka Pranayama, Nadi Shodhana Pranayama. Participants were also engaged in meditation exercises, such as getting quiet, calm, and focused; relaxing, mind slowed down, and staying positive through focusing on things that bring peace to the mind and making efforts to shed the unhelpful thoughts that impede health. Homework assignments were given to the participants at the end of each session and checked at the beginning of each session. Moreover, the therapists discuss good health practices and risk management techniques in and outside the place of work.

Sessions 7–9 involved further disputation and further coaching with conventional yoga practices that could keep the participants’ healthy and improve work efficiency. The therapists help the clients to develop a routine of health practices and positive psychology within their workplace. Clients were taught and led to practice time management skills. Asana poses were also practiced after each session. One hour each was used for CBT and yoga practices.

In weeks 10–11, further helping the participant develop skills for stress management and healthy thoughts as well as yoga exercises (posture, breathing, and meditation exercises) were the major activities. The participants were led toward developing problem-solving, rational thinking, and stress management skills necessary for maintaining a healthy relationship with jobs. Further yoga exercises were practiced after CBT sessions 10 and 11.

#### Phase 3 conclusion and revision

At week 12, participants were supported to ask their questions and clarify personal experiences and life experiences. They shared useful improvements in stress management skills to strengthen strategy use. Yoga exercises- ananas poses, breathing and meditation exercises were practiced. The intervention ended at week 12, and at week 14, post-test evaluation was conducted and the Time 2 data was collected. After 3 months of post-test, a follow-up meeting was held, and follow-up (Time 3) data collection was conducted.

### Recruitment, response rates, attrition, and adherence

Out of the 125 screened prospective participants, 93 met the inclusion criteria and were included in the study. In essence, there was a high response and adherence rate and a low attrition rate. The low attrition rate could be because the researchers offered the participants transport fair for each meeting 91 (97.85%) of the 93 participants who were included in the study completed the three evaluations (pre, post, and follow-up evaluations). Two participants (2.15%) were lost to follow-up for reasons not known to the researchers. One participant in the waitlisted group did not complete the compensatory therapy.

### Study setting

The study was conducted within the university. While CBT took place in a lecture hall, yoga practice sessions were conducted in the university stadium, where most of the recreational and physical exercises by staff and students of the university take place on Saturdays. Randomization and assessment sessions were also conducted in the school hall where CBT interventions were conducted. The sessions were held in one of the institutions that is more centrally situated and convenient to the researchers and participants.

### Data analyses

We used *t*-test statistics to analyze the baseline data, and a 2-way analysis of variance (ANOVA) with repeated measures to compare baseline, post-test, and follow-up data. We used the partial Eta squares to report the effect size of the intervention. We used a paired sample *t*-test to compare participants’ ratings across times 1 and 2, as well as Time 2 and 3. The interaction effect of group × Time was ascertained using 2 × 3 Analysis of Variance (ANOVA) statistics. Bar charts were also used to demonstrate results. Statistical Package for Social Sciences (SPSS) version 24.0 and Microsoft Excel were used for analyses. All results were presented in tables and charts.

## Results

Table 2 shows the *t*-test statistics of differences in all stress subscales between the Y-CBT group and wait-list group (WLG) at baseline data (Time 1). There were no significant variations in the mean scores of the Y-CBT and the WLG participants in the subscales of stress sources perception: Time Management; Work Related Stressors; Professional Distress; Discipline and Motivation; Professional Investment (*p* > 0.001 in each). The total perception of SS scores of Y-CBT groups and WLG did not vary significantly (*p* > 0.001). These indicate that participants in both the Y-CBT group and WLG had an equally high baseline perception of job stressors. Considering the stress manifestation (SM) subscales, Table 2 further shows that both the Y-CBT group and WLG had a non-significant difference in their Stress Manifestation subscales: emotional manifestation, fatigue; cardiovascular manifestation, gastrointestinal behavioral manifestations of stress at baseline (*p* > 0.05 in each case). On the whole, participants in both Y-CBT and WLC groups did not vary significantly in their total TSI rating (*p* > 0.001). The high scores recorded in both groups indicated that participants in both groups not only perceived their jobs as stressful but also experienced both psycho-emotional and physiological symptoms associated with stress (see Table 2).

**TABLE 2:** *T*-test analysis of the baseline data on teachers’ stress inventory subscales.

Subscales	Group	N	$\bar{X}$ ± SD	Df	T	*P*	Mean Diff	95% CILower	Upper
Time management	Y-CBT	46	26.91 ± 3.21	89, 70.964	0.75	0.452	0.71	−1.16	2.59
	Waitlist control	45	26.20 ± 5.45						
Work related stressors	Y-CBT	46	21.17 ± 3.28	89, 88.076	0.40	0.670	0.20	−0.63	2.22
	Waitlist control	45	21.37 ± 3.55						
Professional distress	Y-CBT	46	17.58 ± 2.48	89, 87.542	0.24	0.595	0.37	−0.46	1.72
	Waitlist control	45	17.95 ± 2.76						
Discipline and motivation	Y-CBT	46	21.52 ± 1.73	89, 63.178	0.21	0.628	0.32	−0.46	1.90
	Waitlist control	45	21.80 ± 3.59						
Professional investment	Y-CBT	46	13.20 ± 2.49	89, 88.310	0.85	0.397	0.43	−0.57	1.43
	Waitlist control	45	13.63 ± 2.33						
SS score	Y-CBT	46	101.38 ± 13.23	89, 88.845	0.68	0.561	0.42	−0.82	9.86
	Waitlist control	45	100.97 ± 12.41						
Emotional manifestation	Y-CBT	46	17.78 ± 2.45	89, 79.221	0.18	0.855	0.11	−1.13	1.37
	Waitlist control	45	17.66 ± 3.46						
Fatigue	Y-CBT	46	20.95 ± 1.99	89, 73.019	0.43	0.666	0.24	−0.88	1.37
	Waitlist control	45	20.71 ± 3.23						
Cardiovascular manifestation	Y-CBT	46	9.60 ± 2.04	89, 84.972	0.01	0.986	0.01	−0.94	0.96
	Waitlist control	45	9.60 ± 2.49						
Gastrointestinal manifestation	Y-CBT	46	7.91 ± 2.00	89, 79.445	-1.16	0.248	−0.59	−1.62	0.42
	Waitlist control	45	8.51 ± 2.81						
Behavioral manifestation	Y-CBT	46	18.04 ± 2.34	89, 83.377	-0.00	0.999	−0.00	−1.12	1.12
	Waitlist control	45	18.04 ± 2.99						
SM score	Y-CBT	46	74.30 ± 8.50	89, 1.611	-0.10	0.914	−0.22	−4.41	3.95
	Waitlist control	45	74.53 ± 11.33						
TSI score	Y-CBT	46	175.67 ± 21.92	89, 85.713	-0.01	0.991	−0.07	−1.79	0.93
	Waitlist control	45	175.51 ± 22.73						

SS, stress sources; SM, stress manifestation; TSI, teachers’ stress inventory; $\bar{X}$, mean; SD, standard deviation; d, degree of freedom; t, *t*-test statistic; p, probability value; CI, confidence interval.

Table 3 shows the repeated measures ANOVA for the effect of the Y-CBT on participants’ post-test (Time 2), and follow-up (Time 3). The results show that there were significant main effects of Y-CBT on all subscales of stress sources at post-test evaluation and follow-up. Participants in the Y-CBT and WLG groups varied significantly (*p* < 0.001) in their time management at Time 2, with the Y-CBT rating lower than the WLG. The Y-CBT group rated significantly lower than the WLG at the Time 3 evaluation (*p* < 0.001), indicating that the effect of the Y-CBT on participants’ time management was sustained through a 3 months follow-up. The eta squared: η^2^ = 0.40 and 0.44 at Time 2 and 3, respectively, indicated moderate effect size. The work-related stressors mean scores of participants in the Y-CBT group and WLG had a significant difference (*p* < 0.001) which was sustained through the 3 months follow-up (*p* < 0.001), with a relatively high effect size. Furthermore, in the professional distress subscale, the Y-CBT group also rated significantly lower than the WLG at time 2 and 3 evaluations (*p* < 0.001 in each case).

**TABLE 3:** Repeated measures ANOVA for post-test and Follow-up evaluation.

Subscales	Time	Y-CBT (*N* = 46	Waitlist control group (*n* = 44)	Df	F	Sig	95% CI	η^2^
Time management	Time 2	16.34 ± 7.74	27.77 ± 6.04	1, 89	61.363	0.000	−14.32, −8.53	0.40
	Time 3	16.58 ± 9.18	28.97 ± 3.72	1, 89	70.48	0.000	−15.32, −9.45	0.44
Work related stressors	Time 2	12.50 ± 6.54	20.37 ± 4.61	1, 89	43.867	0.000	−10.24, −5.51	0.33
	Time 3	11.86 ± 7.01	21.22 ± 2.97	1, 89	68.03	0.000	−11.60, −7.09	0.43
Professional distress	Time 2	8.19 ± 5.08	17.22 ± 3.54	1, 89	96.183	0.000	−10.85, −7.19	0.51
	Time 3	10.23 ± 5.77	17.93 ± 1.86	1, 89	72.41	0.000	−9.49, −589	0.44
Discipline and motivation	Time 2	10.89 ± 6.08	21.28 ± 3.29	1, 89	102.003	0.000	−12.44, −8.35	0.53
	Time 3	11.86 ± 6.69	21.73 ± 2.56	1, 89	85.34	0.000	−11.98, −7.74	0.49
Professional investment	Time 2	7.15 ± 4.16	13.95 ± 2.78	1, 89	83.529	0.000	−8.28, −5.32	0.48
	Time 3	8.28 ± 4.60	14.31 ± 2.49	1, 89	59.86	0.000	−7.57, −4.48	0.40
SS score	Time 2	55.08 ± 27.34	100.62 ± 19.31	1, 89	83.850	0.000	−55.41, −0.35	0.48
	Time 3	58.84 ± 32.62	104.17 ± 12.26	1, 89	76.30	0.000	−55.64, −35.01	0.46
Emotional manifestation	Time 2	7.30 ± 1.99	17.42 ± 3.00	1, 89	359.364	0.000	−11.17, −9.05	0.80
	Time 3	8.21 ± 2.86	17.82 ± 2.47	1, 89	192.55	0.000	−10.72, −8.48	0.76
Fatigue	Time 2	7.06 ± 1.69	17.13 ± 3.14	1, 89	363.859	0.000	−11.11, −9.01	0.80
	Time 3	8.34 ± 2.52	17.57 ± 2.51	1, 89	305.03	0.000	−10.28, −8.17	0.77
Cardiovascular manifestation	Time 2	4.21 ± 1.17	10.55 ± 1.77	1, 89	404.749	0.000	−6.96, −5.17	0.82
	Time 3	5.54 ± 2.28	10.77 ± 1.41	1, 89	171.62	0.000	−6.02, −4.44	0.65
Gastrointestinal manifestation	Time 2	10.21 ± 1.69	10.06 ± 2.22	1, 89	0.132	0.717	−0.67, −0.97	0.00
	Time 3	4.56 ± 1.60	10.77 ± 1.83	1, 89	297.00	0.000	−6.92 −5.49	0.76
Behavioral manifestation	Time 2	5.34 ± 1.19	13.60 ± 2.98	1, 89	302.845	0.000	−9.19, −7.30	0.77
	Time 3	6.69 ± 2.79	14.24 ± 1.87	89	227.97	0.000	−8.54, −6.55	0.71
SM score	Time 2	34.15 ± 5.24	68.77 ± 10.63	1, 89	390.627	0.000	−38.10, −0.31	0.81
Global TSI score	Time 3	33.36 ± 9.73	71.20 ± 9.06	1, 89	367.50	0.000	−41.75,−33.90	0.80
	Time 2	89.23 ± 25.65	169.39 ± 21.67	1, 89	310.472	0.000	−88.11, −0.41	0.78
	Time 3	92.21 ± 29.73	175.39 ± 9.06	1, 89	305.760	0.000	−91.75,−38.90	0.70

SS, stress sources; SM, stress manifestation; TSI, teachers’ stress inventory; $\bar{X}$, mean; SD, standard deviation; df, degree of freedom; F, analysis of variance test statistic; p, probability value; CI, confidence interval and η^2^ = Partial Eta square (effect size).

Additionally, significant differences were recorded in the discipline and motivation subscale between the groups at post-test and follow-up (*p* < 0.001). In the professional investment subscale, the Y-CBT and WLG recorded significant differences at Time 2 and 3 (*p* = 0.000 in each). The global perception of a stressor as measured by total stress sources scores recorded significant differences between Y-CBT and WLG at Time 2 and Time 3 (*p* < 0.001). These results show that the participants’ negative perception of job stressors was reduced using Y-CBT intervention modalities.

Considering the SM subscales in Table 3, the Y-CBT group showed significantly reduced scores than the WLG in Emotional Manifestation, fatigue, cardiovascular symptoms, gastrointestinal symptoms, and behavioral manifestation (*p* < 0.001 in each case), with high effect size. The total stress manifestation score was significantly reduced in the Y-CBT group over the WLG at post-test (*p* < 0.001), and at follow-up (*p* < 0.001). Cumulatively, a significant difference was found in the total TSI score of the Y-CBT group and WLG at post-test (*p* < 0.001), and at follow-up (*p* < 0.001). These results indicate that Y-CBT led to a sustained reducing effect on all dimensions of the global stress of university teachers (see Table 3). In summary, these results suggest a significant decline in stress level following Y-CBT intervention.

**FIGURE 3:**
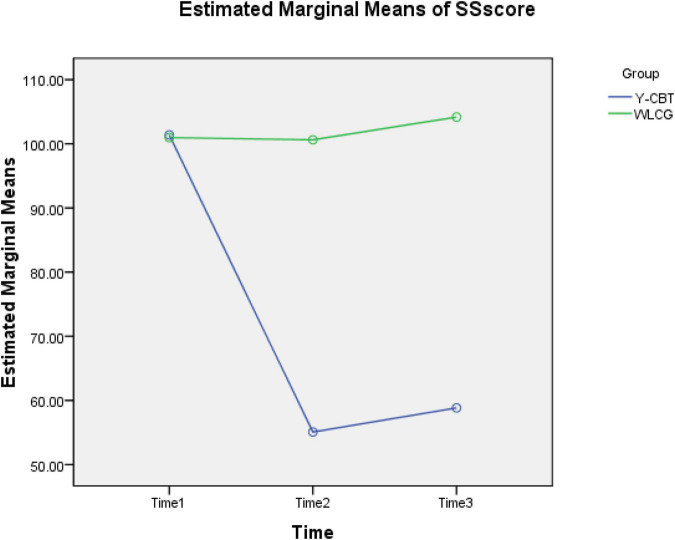
Interaction effect of time and intervention on participants’ SS scores.

Furthermore, we conducted a paired sample *t*-tests to explore changes in the three main subscale scores (stress sources, stress manifestation, and global stress scores) across baseline, post-test, and follow-up. Paired sample *t*-test result showed that SS scores of Y-CBT group decreased significantly between Time 1 and 2 [*t* (89) = 11.45, *p* < 0.001, CI = 0.37.69, 53.78] and non-significantly between Time 2 and 3 [*t* (89) = −0.626, *p* = 0.53, CI = 6.00, −15.85]. Contrarily, WLG did not have significant change across Time 1–2 [*t* (89) = −0.954, *p* = 0.345, CI = −0.9.61, 3.43] and Time 2–3 [*t* (89) = −7.68, *p* = 0.19, CI = −7.68, 0.57]. These show a sustained reduction in SS score following Y-CBT intervention (see Figure 3).

**FIGURE 4:**
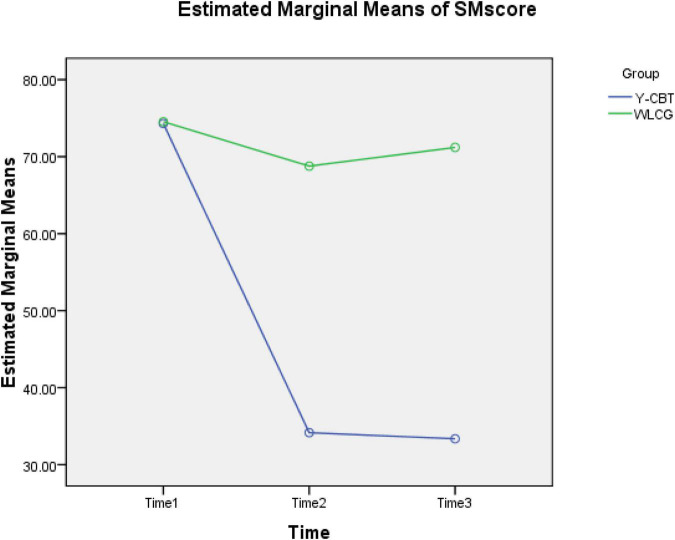
Interaction effect of time and intervention on participants’ SM scores.

Regarding SM, there was also significant decrease across Time 1 and 2 [*t* (89) = 22.60, *p* < 0.001, CI = 32.02, 38.28] but non-significant differences in Time 2 and 3 [*t* (89) = −555, *p* = 0.582, CI = −2.05, 3.62] for Y-CBT group. Contrarily, participants in the WLC group recorded non-significant difference in SM scores across Time 1–2 [*t* (89) = 0.67, *p* = 0.506, CI = −2.54, 5.07] and 2–3 [*t* (89) = −1.58, *p* = 0.110, CI = −7.20, 0.85]. This suggests that the reduced stress manifestation at post-test was a product of the interaction effect of the coaching intervention and time, and was sustained (see Figure 4).

**FIGURE 5:**
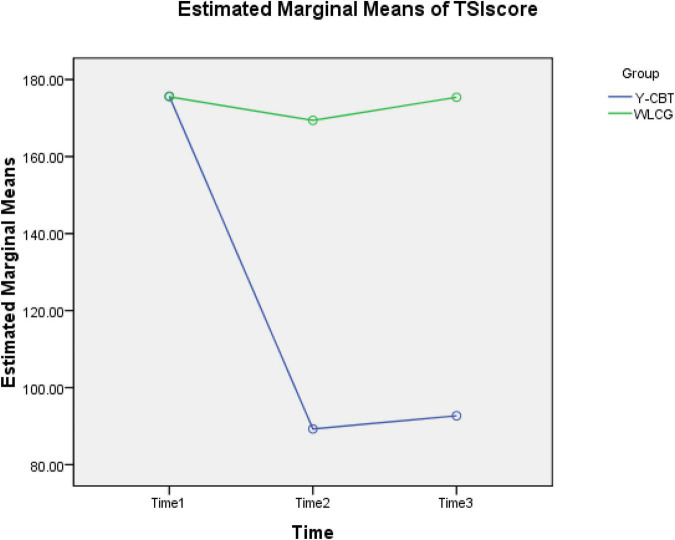
Interaction effect of time and intervention on participants’ TSI scores.

**FIGURE 6:**
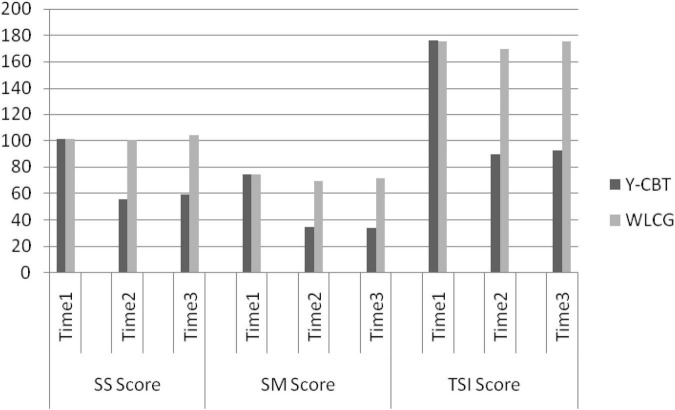
Interaction effect of Time and intervention on Participants’ SS, SM, and TSI scores on the three measurements.

Regarding the global TSI score, the Y-CBT participants also had significant reduction in across Time 1 and 2 [*t* (89) = 16.892, *p* = 0.000, CI = 71.24, 90.13]; which did not change significantly across Time 2–3 [*t* (89) = −0.444, *p* = 0.659, CI = −16.48, 10.52]. On the other hand, participants in the WLC group did not record significant changes in their TSI scores across Time 1–2 [*t* (89) = −0.480, *p* = 0.633, CI = −12.12, −7.46] and Time 2–3 [*t* (89) = −2.09, *p* = 0.550, CI = −12.16, 0.20] (Figures 5, 6). These outcomes indicate that TSI scores of university lecturers reduced significantly following Y-CBT intervention.

### Scores on the three measurements

Figure 6 shows an excerpt of the result of 2 × 3 Analysis of Variance (ANOVA) statistics for the interaction effect of group × Time on SS, SM, and TSI dimensions. At baseline (Time 1), the Y-CBT and waitlist groups had no significant difference in their stress sources (SS), stress manifestation, and global stress scores, but there were significant differences in SS and SM, as well as the global score (TSI) at post-test (Time 2) and follow-up (Time 3). These suggest that all reductions in the outcome variables are attributable to the intervention, and not due to time changes.

## Discussion

Job stress has been found to be highly prevalent among university teachers, implicated in stress-induced health issues, undermined work productivity, deficiency in coping with work demand, and consideration of job change. In this study, we examined the effectiveness of Y-CBT in managing the perception of job stress and stressors among university academic staff (teachers). Results demonstrate that while Y-CBT and wait-list groups (WLG) had high, and relatively equal levels of stress at baseline evaluation, all stress dimensions of the Y-CBT group reduced following intervention. The results further demonstrated the interaction effects of Time and intervention on the measures of participants’ stress, suggesting that the decrease in stress scores across time was strictly owing to Y-CBT intercession and not due to changes in time. Given the significant decrease in participants’ stress, it can be inferred that through Y-CBT, lecturers could be helped to change their reactions to stressful experiences and minimize related physiological and behavioral symptoms, even in demanding and challenging work conditions.

This outcome is intriguing as it supports a few other studies in the field of research in using complementary approaches in positive psychology for maintaining a healthy outlook in the workplace, and improving psycho-physiological outcomes (Froman, 2010; Orkibi and Brandt, 2015; Bayer, 2018; Donaldson et al., 2019; Lianov et al., 2020). Prior intervention studies have also noted the efficacy of Y-CBT programs in minimizing psychosomatics disorders. For example, Allen et al. (2018) investigated the efficacy of 12-weeks Y-CBT in a pilot study on signs of pain in youth and found that Y-CBT can be used to reduce psychosomatic symptoms. In a related study, Khalsa et al. (2015) found that Y-CBT leads to significant reductions in anxiety, depressive symptoms, panic, sleep disorders, and the quality of life of participants.

The mechanism of change in Y-CBT follows (i) disputation (D), which modifies the pessimistic and dysfunctional viewpoint “B” regarding the demanding condition “A” and substituting them with the positive and helpful ones (E); (ii) building problem-solving skills required to contend with stress sources; and (iii) using physical activities to harmonize the mind and body and overcome distrustful psychosomatic and cognitive symptoms emanating from job-demands (Nakao, 2010; Ganapathi and Panchanatham, 2012). Through self-monitoring, Y-CBT helps participants reexamine the dexterity that furnishes them to realize their own thinking regarding the current work condition for purposeful efforts. Y-CBT participants can apply the developed skills for stress management and for a new practical approach to job demand (Michie, 2002). Prior studies suggest that a constructive transformation in stress perception can translate to a decrease in physiological and psychological indicators of job stress (Michie, 2002; Stults-Kolehmainen and Sinha, 2014; Havermans et al., 2018). Through Y-CBT, the stress perception of the participant changes, leading to a problem-focused approach to stress management, reducing the associated anxiety, depression, and musculoskeletal symptoms. Khalsa et al. (2015) showed a significant reduction in generalized anxiety with Y-CBT. This could be because Y-CBT resists negative thoughts, feelings, and emotions associated with work stressors (occupational environments), substituting them with more helpful ones, thereby dealing with emotional and somatic symptoms that put the lecturers in a vicious circle of stress reactions and pain (Mohamed, 2017; Bayer, 2018).

The effectiveness of Y-CBT could also be partly due to its characteristic strategy of combining yoga postures and regulation of breath repeatedly appealing to relaxation leading to reduced stress activation and hypothalamic-pituitary axis activity (Khalsa et al., 2015). Hence, such strategies particularly target maladaptive cognitive processes that characterize stress. Moreover, both yoga and CBT have the capacity to minimize irrational belief processes and fill thoughts with positivity. While CBT helps clients to recognize, oppose, and substitute automatic judgments with more efficient ones, yoga and meditation restrain the inclination toward negative thoughts by minimizing the default mode network (DMN) and making the individual focus on physical activities (Khalsa et al., 2015). Physical exercise is able to create a synergy between mind and body to bring about a positive shift in cognitive, emotional, and physiological wellbeing. By this, yoga and CBT complement each other to lessen stress, both by plummeting the propensity for an unhelpful view to arise (yoga) and by substituting the negative thoughts that do arise (CBT).

Reducing workplace stress reduces psychopathological symptoms such as headache, anxiety, and musculoskeletal problems (Rao and Chandraiah, 2012; Havermans et al., 2018) which could undermine employees’ effectiveness. As such, the reduction of stress in university teachers could minimize negative health conditions and keep them healthy and stable to face the tasks of teaching and research. The present study yields itself to a gray area that addresses the present need of Nigerian society. The intervention is considered well-timed, given the heightened stress among university lecturers in federal universities who experience a high demand for teaching and research, together with stagnation due to the use of Thomson Reuters (impact factor) journal publications as the benchmark for promotion (Ibegbulam et al., 2017; Okoye and Okoye, 2018). The study productively confirms the efficacy of Y-CBT in reducing negative perceptions and emotions associated with job stress as well as the accompanying physiological symptoms among university teachers, using a randomized control trial.

Though randomized control trial research on Y-CBT is still emerging, the therapeutic modality can be applied to different populations. For instance, when Khalsa et al. (2015) used Y-CBT for the treatment of generalized anxiety disorder in resistant clients at a community mental health clinic, the intervention was found effective. With the present study still confirming the efficacy of Y-CBT in a non-clinical group setting, it is likely that the Y-CBT intervention is effective across contexts. Additionally, the modality can be valuable in treating other mental health conditions that lead to rumination (Hasenkamp et al., 2012). Hence, more studies are needed to test the efficacy of Y-CBT in general improvement of wellbeing and treating stress, depression, and anxiety across diverse populations.

### Limitations and directions for future studies

This study may be limited by a relatively small sample, which threatens the generalization of the results to other contexts. Future studies should use a larger sample to substantiate Y-CBT for stress management in university lecturers. This study collected data based only on self-report measures. Further studies could utilize added qualitative methods to ascertain participants’ satisfaction with therapy and therapists. The Y-CBT package could be validated in a different population of workers who experience stress. Future studies may further compare Y-CBT and conventional CBT as the present study failed to explore that area.

Assigning individuals to a waiting control group could present an ethical dilemma. It is possible for waitlisted individuals to deteriorate during the waiting period. Future research may utilize treatment as usual for the control group to provide an adequate comparison. By allowing the control group to participate in the same intervention, the waitlist control trial design preserved the concept of justice. In this study, the waitlist control trial design may be problematic due to an overestimation of the effects of the intervention. It is unclear whether the condition of the control group improved equally following the compensating intervention. Future studies could assess the waitlist after the compensatory intervention to determine whether or not the compensation was effective. Additionally, it would have been more specific to use multiple treatment groups that can be compared to a control condition. A more robust intervention study can assign participants to Yoga alone, CBT alone, combine Yoda and CBT, and control conditions using Solomon Four-Group design to fully identify what the key ingredients might be accountable for positive outcomes.

### Practical implications

The findings of the current study have practical value for practitioners such as positive psychologists, counseling psychologists, therapists, and future researchers. The study has indicated that Y-CBT can help university lecturers to develop a more positive outlook toward stressful situations. Positive psychology can leverage on this framework to improve human wellbeing, life satisfaction, and resilience, especially in challenging occupational environments. Counselors and therapists can use the Y-CBT framework to build coping strategies in their clients, especially for coping with stress. Nourishing the mind and body is a better approach to supporting individuals to thrive in a demanding environment, and can be offered through Y-CBT. Counseling psychologists and therapists can apply Y-CBT in the treatment of common mental health issues that interfere with daily activities. Furthermore, future researchers can use Y-CBT in other populations for treating stress and related mental health conditions such as anxiety, and depression.

## Conclusion of the study

This study examined the efficacy of a 12-week Y-CBT in managing work stress among university lecturers. Participants in the Y-CBT showed a significantly reduced in all dimensions of stress (sources, stress manifestations, and global stress inventory scores) over those who were waitlisted. It is, therefore, concluded that Y-CBT is efficacious in deflating stress among university teachers. On this groundwork, more studies could further appraise Y-CBT in other populations of employees who experience occupational stress.

## Data availability statement

The raw data supporting the conclusions of this article will be made available by the authors, without undue reservation.

## Ethics statement

The studies involving human participants were reviewed and approved by the Faculty of Educational Research Ethics Committees University of Nigeria, Nsukka and Alex Ekwueme Federal University Ndufu-Alike Nigeria. The patients/participants provided their written informed consent to participate in this study.

## Author contributions

MB, FO, and JU conceptualized the research topic and wrote the draft. CE and EN conducted the intervention and collected data. DE, NI, and UE analyzed and interpreted the data. All authors proofread the manuscript before submission.

## References

[B1] Akinmayowa J. T. Kadiri A. P. (2014). Stress among academic staff in a Nigerian university. *Covenant J. Bus. Soc. Sci.* 65 73–89.

[B2] Ali K. K. Basavarjappa B. Nik M. M. (2013). Effectiveness of cognitive behavioral therapy on depression among high school students. *J. Appl. Res.* 3 147–158.

[B3] Allen T. M. Wren A. A. Anderson L. M. Sabholk A. Mauro C. F. (2018). A group CBT-yoga protocol targeting pain-related and internalizing symptoms in youth. *Clin. Pract. Pediatr. Psychol.* 6 7–18. 10.1037/cpp0000206

[B4] American psychological Association (2012). *Report Highlights: Stress in America: Paying for our Health.* Washington, DC: American psychological Association.

[B5] American Psychological Association (2017). *Ethical principles of psychologists and code of conduct. Including 2010 and 2016 amendments.* Available online at: https://www.apa.org/ethics/code/index (accessed 2002).

[B6] Arapovic-Johansson B. (2020). *Stress Prevention at Work: Intervention Effectiveness and Implementation Process Evaluation.* Doctoral dissertation. Sweden: Karolinska Institutet.

[B7] Archibong J. A. Bassey A. O. Effiom D. O. (2010). Occupational stress sources among university academic staff. *Eur. J. Educ. Stud.* 2 217–225.

[B8] Arokium J. (2010). *An investigation of the coping strategies used by teachers to deal with stress.* Doctoral dissertation. Durban: University of Kwa-Zulu Natal.

[B9] Azeem S. M. Nazir N. A. (2008). A study of job burnout among university teachers. *Psychol. Dev. Soc.* 20 51–64. 10.1177/097133360702000103

[B10] Ballas P. (2009). *Depression Overview.* Baltimore, MD: University of Maryland & Medical Center.

[B11] Barmby P. (2006). Improving teacher recruitment and retention: the importance of workload and pupil behaviour. *Educ. Res.* 48 247–265. 10.1080/00131880600732314

[B12] Bayer J. L. (2018). *An Eight-Week Forrest Yoga Intervention for Chronic Pain: Effect on Pain Interference, Pain Severity, and Psychological Outcomes.* Doctoral dissertation. Iowa City, IA: University of Iowa.

[B13] Blix A. G. Cruise R. J. Mitchell B. M. Blix G. G. (1994). Occupational stress among university teachers. *Educ. Res.* 36 157–169. 10.1080/0013188940360205

[B14] Boshoff S. M. (2011). *Validation of the Teacher Stress Inventory (TSI) in a South African Context: The SABPA Study.* Doctoral dissertation. North-West University.

[B15] Bragard I. Etienne A. M. Faymonville M. E. Coucke P. Lifrange E. Schroeder H. (2017). A nonrandomized comparison study of self-hypnosis, yoga, and cognitive-behavioral therapy to reduce emotional distress in breast cancer patients. *Int. J. Clin. Exp. Hypnosis* 65 189–209. 10.1080/00207144.2017.1276363 28230462

[B16] Brenes G. A. Divers J. Miller M. E. Danhauer S. C. (2018). A randomized preference trial of cognitive-behavioral therapy and yoga for the treatment of worry in anxious older adults. *Contemp. Clin. Trials Commun.* 10 169–176. 10.1016/j.conctc.2018.05.002 30009275 PMC6042466

[B17] Bridges L. Sharma M. (2017). The efficacy of yoga as a form of treatment for depression. *J. Eviden. Based Complement. Altern. Med.* 22 1017–1028. 10.1177/2156587217715927 28664775 PMC5871291

[B18] Brown E. L. Valenti M. W. (2013). Merging pathways: the interdisciplinary study of emotional labor and therapeutic alliances in schools. *Commu. Psychol.* 46 35–37. 10.1037/e558112013-011

[B19] Busari A. O. (2018). Motivation, stress, anxiety and emotions as predictors of academic boredom among degree students of National Teachers’ Institute Osogbo, Osun State, Nigeria. *World Sci. News* 112 165–179.

[B20] Butera R. Byron E. Elgelid S. (2015). *Yoga Therapy for Stress and Anxiety: Create a Personalized Holistic Plan to Balance Your Life.* Woodbury, MN: Llewellyn Worldwide.

[B21] Camacho D. (2017). *Applying a Cognitive-Behavioral Model to Conceptualize Burnout and Coping for Teachers in Urban Schools.* Dissertation. Chicago, IL: Loyola University Chicago.

[B22] Chong C. S. Tsunaka M. Chan E. P. (2011). Effects of yoga on stress management in healthy adults: a systematic review. *Altern. Ther. Health Med.* 17 32–38. 21614942

[B23] Cramer H. Ward L. Steel A. Lauche R. Dobos G. Zhang Y. (2016). Prevalence, patterns and predictors of yoga use: results of a US nationally representative survey. *Am. J. Prevent. Med.* 50 230–235. 10.1016/j.amepre.2015.07.037 26497261

[B24] Darling-Hammond L. (2010). Teacher education and the American future. *J. Teach. Educ.* 61 35–47. 10.1177/0022487109348024

[B25] David D. (2015). “Rational emotive behavior therapy (REBT),” in *Encyclopedia of Clinical Psychology*, eds Cautin R. L. Lilienfeld S. O. (New Jersey: Wiley-Blackwell). 10.1002/9781118625392.wbecp077

[B26] David D. Szentagotai A. (2006). Cognitions in cognitive-behavioral psychotherapies; toward an integrative model. *Clin. Psychol. Rev.* 26 284–298. 10.1016/j.cpr.2005.09.003 16325974

[B27] De Neve J. E. Diener E. Tay L. Xuerb C. (2013). “The objective benefits of subjective well-being,” in *World Happiness Report 2013*, eds Helliwell J. Layard R. Sachs J. (New York, NY: UN Sustainable 10 Development Solution Network).

[B28] Deb S. Strodl E. Sun J. (2015). Academic stress, parental pressure, anxiety and mental health among Indian high school students. *Int. J. Psychol. Behav. Sci.* 5 26–34.

[B29] Desveaux L. Agarwal P. Shaw J. Hensel J. M. Mukerji G. Onabajo N. (2016). A randomized wait-list control trial to evaluate the impact of a mobile application to improve self-management of individuals with type 2 diabetes: a study protocol. *BMC Med. Inform. Decis. Mak.* 16:144. 10.1186/s12911-016-0381-5 27842539 PMC5109669

[B30] DiGiuseppe R. A. Doyle K. A. Dryden W. Backx W. (2014). *A Practitioner’s Guide to Rational-Emotive Behavior Therapy.* New York, NY: Oxford University Press. 10.1093/med:psych/9780199743049.001.0001

[B31] Dike I. C. Onyishi C. N. Adimora D. E. Ugodulunwa C. A. Adama G. N. Ugwu G. C. (2021). Yoga complemented cognitive behavioral therapy on job burnout among teachers of children with autism spectrum disorders. *Medicine* 100:e25801. 10.1097/MD.0000000000025801 34087823 PMC8183729

[B32] Dobson K. S. Dozois D. J. (2010). *Historical and Philosophical Bases of the Cognitive-Behavioral Therapies.* New York, NY: Guilford Press.

[B33] Donaldson S. I. Lee J. Y. Donaldson S. I. (2019). “The effectiveness of positive psychology interventions in the workplace: a theory-driven evaluation approach,” in *Theoretical Approaches to Multi-Cultural Positive Psychological Interventions*, eds Van Zyl L. E. Rothmann S. (Berlin: Springer), 115–159. 10.1007/978-3-030-20583-6_6

[B34] Dore J. (2016). *Q & A: Yoga-enhanced cognitive behavioral therapy.* Available online at: https://pro.psychcentral.com/q-a-yoga-enhanced-cognitivebehavioral-therapy/ (accessed December 5, 2020).

[B35] Ekundayo H. T. Kolawole A. O. (2013). Stress among secondary school teachers in Ekiti State, Nigeria. *J. Educ. Soc. Res.* 3 311–311. 10.5901/jesr.2013.v3n2p311

[B36] Ellis A. (1995). Changing rational-emotive therapy (RET) to rational emotive behavior therapy (REBT). *J. Ration. -Emot. Cogn. Behav. Ther.* 13 85–89. 10.1007/BF02354453 36035437

[B37] Eskandar R. Sasan B. (2015). Effectsof yoga on anxiety and depression in women. *Br. J. Sports Med.* 44 (Suppl.), i1–i182. 10.1136/bjsm.2010.078725.227

[B38] European Commission (2000). *Guideline on Work Related Stress. Spice of Life or Kiss of Death?.* Luxembourg: Office for official publications of the European communities.

[B39] Fimian M. J. (1984). The development of an instrument to measure occupational stress in teachers: the teacher stress inventory. *J. Occup. Psychol.* 57 277–293. 10.1111/j.2044-8325.1984.tb00169.x

[B40] Fimian M. J. Fastenau P. S. (1990). The validity and reliability of the teacher stress inventory: A re-analysis of aggregate data. *J. Organ. Behav.* 11, 151–157. 10.1002/job.4030110206

[B41] Fisher M. H. (2011). Factors influencing stress, burnout & retention of secondary school teachers. *Curr. Issues Educ.* 14, 1–37.

[B42] Forfylow A. L. (2011). Integrating yoga with psychotherapy: a complementary treatment for anxiety and depression. *Can. J. Counsel. Psychother.* 45 132–150.

[B43] Froman L. (2010). Positive psychology in the workplace. *J. Adult Dev.* 17 59–69. 10.1007/s10804-009-9080-0

[B44] Ganapathi N. Panchanatham N. (2012). Workplace stress: the need for communication and knowledge sharing. *Internat. J. Exclusive Manage Res.* 2, 1–15.

[B45] Gold Y. Roth R. A. (2013). *Teachers Managing Stress & Preventing Burnout.* London: Routledge. 10.4324/9780203209899

[B46] General Assembly of the World Medical Association (2014). World medical association declaration of Helsinki: ethical principles for medical research involving human subjects. *J. Am. College Dent.* 81 14–18 25951678

[B47] Granath J. Ingvarsson S. von Thiele U. Lundberg U. (2006). Stress management: a randomized study of cognitive behavioural therapy and yoga. *Cogn. Behav. Ther.* 35 3–10. 10.1080/16506070500401292 16500773

[B48] Grensman A. Acharya B. D. Wändell P. Nilsson G. H. Falkenberg T. Sundin Ö (2018). Effect of traditional yoga, mindfulness–based cognitive therapy, and cognitive behavioral therapy, on health related quality of life: a randomized controlled trial on patients on sick leave because of burnout. *BMC Complement. Altern. Med.* 18:80. 10.1186/s12906-018-2141-9 29510704 PMC5839058

[B49] Halliwell E. Jarman H. Tylka T. L. Slater A. (2018). Evaluating the impact of a brief yoga intervention on preadolescents’ body image and mood. *Body Image* 27 196–201. 10.1016/j.bodyim.2018.10.003 30359869

[B50] Harish K. A. JeyaPrabha B. (2018). An empirical study on the stressors of teachers and its impact on occupational stress and job satisfaction of teachers in government and private sectors. *Int. J. Pure Appl. Math.* 118 689–698.

[B51] Hasenkamp W. Wilson-Mendenhall C. D. Duncan E. Barsalou L. W. (2012). Mind wandering and attention during focused meditation: a fine-grained temporal analysis of fluctuating cognitive states. *Neuroimage* 59 750–760. 10.1016/j.neuroimage.2011.07.008 21782031

[B52] Hassard J. Teo K. Cox T. Dewe P. Cosmar M. Grundler R. (2014). *Calculating the cost of work-related stress & psychosocial risks. A literature reviews.* Luxembourg: European Agency for Safety & health at work (EUOSHA), 1–40. Available online at: https://eprints.bbk.ac.uk/id/eprint/20923/1/2014%20EUOSHA.%20Calculating%20the%20cos (accessed December 14, 2022).

[B53] Havermans B. M. Brouwers E. P. Hoek R. J. Anema J. R. van der Beek A. J. Boot C. R. (2018). Work stress prevention needs of employees and supervisors. *BMC Public Health* 18:642. 10.1186/s12889-018-5535-1 29784044 PMC5963034

[B54] Ibegbulam I. J. Uzoagba N. Igbo U. (2017). Academic staff views on the use and challenges of impact factor for assessment for promotion and career progression. *J. Appl. Inf. Sci. Technol.* 10, 126–134.

[B55] Ingersoll R. M. (2001). Teacher turnover and teacher shortages: an organizational analysis. *Am. Educ. Res. J.* 38 499–534. 10.3102/00028312038003499 19954868

[B56] Jasmine E. (2010). *The Impact of Cognitive Behavior Therapy on Irrational Beliefs, Self-Esteem, Self-Acceptance and Depression Among Late Adolescents.* Mysore: University of Mysore.

[B57] Kadden R. M. (2001). Behavioral and cognitive–behavioral treatments for alcoholism: research opportunities. *Addict. Behav.* 26 489–507. 10.1016/S0306-4603(00)00139-8 11456073

[B58] Khalsa M. K. Greiner-Ferris J. M. Hofmann S. G. Khalsa S. B. S. (2015). Yoga-enhanced cognitive behavioural therapy (Y-CBT) for anxiety management: a pilot study. *Clin. Psychol. Psychother.* 22 364–371. 10.1002/cpp.1902 24804619 PMC4224639

[B59] Kyriacou C. (2001). Teacher stress: directions for future research. *Educ. Rev.* 53 27–35. 10.1080/00131910120033628

[B60] Lever N. Mathis E. Mayworm A. (2017). School mental health is not just for students: why teacher and school staff wellness matters. *Rep. Emot. Behav. Disord. Youth* 17 6–12. 30705611 PMC6350815

[B61] Li A. Goldsmith C. A. (2012). The effects of yoga on anxiety and stress. *Altern. Med. Rev.* 17 21–35. 22502620

[B62] Li W. Kou C. (2018). Prevalence and correlates of psychological stress among teachers at a national key comprehensive university in China. *Int. J. Occupat. Environ. Health* 24 7–16. 10.1080/10773525.2018.1500803 30047833 PMC6225434

[B63] Lianov L. S. Barron G. C. Fredrickson B. L. Hashmi S. Klemes A. Krishnaswami J. (2020). Positive psychology in health care: defining key stakeholders and their roles. *Transl. Behav. Med.* 10 637–647. 10.1093/tbm/ibz150 32766868

[B64] Maddux R. E. Daukantaite D. Tellhed U. (2018). The effects of yoga on stress and psychological health among employees: an 8- and 16-week intervention. *Anxiety Stress Coping Int. J.* 31 121–134. 10.1080/10615806.2017.1405261 29166771

[B65] Marten W. D. Wilkerson B. (2003). Stress, work and mental health: a global perspective. *Acta Neuropsych.* 15 44–53. 10.1034/j.1601-5215.2003.00017.x 26984706

[B66] Masuku S. Muchemwa S. (2015). Occupational stress among university lecturers: a case of Zimbabwe. *US China Educ. Rev.* 5 258–266. 10.17265/2161-623X/2015.04A.003

[B67] Math S. B. Srinivasaraju R. (2010). Indian Psychiatric epidemiological studies: learning from the past. *Indian J. Psychiatry* 52 (Suppl.1), S95–S103. 10.4103/0019-5545.69220 21836725 PMC3146182

[B68] McCarthy C. J. Lambart R. G. O’Donnell M. Melendres L. T. (2009). The teacher relation of elementary teachers’ experience, stress & coping resources to burnout symptoms. *Elemen. Sch. J.* 109 282–300. 10.1086/592308

[B69] Meng Q. Wang G. (2018). A research on sources of university faculty occupational stress: a chinese case study. *Psychol. Res. Behav. Manag.* 11 597–605. 10.2147/PRBM.S187295 30573995 PMC6292231

[B70] Michie S. (2002). Causes and management of stress at work. *Occup. Environ. Med.* 59 67–72. 10.1136/oem.59.1.67 11836475 PMC1740194

[B71] Milutinović D. Golubović B. Brkić N. Prokeš B. (2012). Professional stress and health among critical care nurses in Serbia. *Arhiv za Higijenu Rada i Toksikologiju* 63 171–179. 10.2478/10004-1254-63-2012-2140 22728799

[B72] Mohamed S. M. (2017). Effect of cognitive behavioral treatment program on anxiety and self-esteem among secondary school students. *Am. J. Nurs.* 6 193–201. 10.11648/j.ajns.20170603.17

[B73] Nagendra H. R. (2013). Integrated yoga therapy for mental illness. *Indian J. Psychiatry* 55 (Suppl. 3), S337–S339. 10.4103/0019-5545.116299 24049195 PMC3768208

[B74] Nakao M. (2010). Work-related stress and psychosomatic medicine. *BioPsychoSocial Med.* 4:4. 10.1186/1751-0759-4-4 20504368 PMC2882896

[B75] Naveen G. H. Thirthalli J. Rao M. G. Varambally S. Christopher R. Gangadhar B. N. (2013). Positive therapeutic and neurotropic effects of yoga in depression: a comparative study. *Indian J. Psychiatry* 55 (Suppl. 3), S400–S404. 10.4103/0019-5545.116313 24049208 PMC3768221

[B76] Nwokeoma B. N. Ede M. O. Nwosu N. Ikechukwu-Illomuanya A. Ogba F. N. Ugwoezuonu A. U. (2019). Impact of rational emotive occupational health coaching on work-related stress management among staff of Nigeria police force. *Medicine* 98:e16724. 10.1097/MD.0000000000016724 31517811 PMC6750331

[B77] Obiweluozo P. E. Dike I. C. Ogba F. N. Elom C. O. Orabueze F. O. Okoye-Ugwu S. (2021). Stress in teachers of children with neuro-developmental disorders: effect of blended rational emotive behavioral therapy. *Sci. Progr.* 104:00368504211050278. 10.1177/00368504211050278 34783626 PMC10402289

[B78] Ofoegbu F. Nwadiani M. (2006). Level of stress among lecturers in Nigerian universities. *J. Industr. Psychol.* 33 66–75.

[B79] Ogba F. N. Ede M. O. Onyishi C. N. Agu P. U. Ikechukwu-Ilomuanya A. B. Igbo J. N. (2019). Effectiveness of music therapy with relaxation technique on stress management as measured by perceived stress scale. *Medicine* 98:e15107. 10.1097/MD.0000000000015107 30985665 PMC6485837

[B80] Ogba F. N. Igu N. C. N. (2012). Impact of strike action on the implementation of academic programmes in nigerian Universities. *Afr. J. Pedag.* 3 13–25.

[B81] Ogbuanya T. C. Eseadi C. Orji C. T. Ohanu I. B. Bakare J. Ede M. O. (2017). Effect of rational emotive behavioral coaching on occupational stress & work ability among electronics workshop instructors in Nigeria. *J. Med.* 96:e6891. 10.1097/MD.0000000000006891 28489795 PMC5428629

[B82] Okeke C. I. O. (2013). An empirical study of stressors that imping on teachers in secondary schools in Swaziland. *S. Afr. J. Educ.* 33 1–12.

[B83] Okeke C. I. O. Dlamini C. C. (2013). An empirical study of stressors that impinge on teachers in secondary schools in Swaziland. *S. Afr. J. Educ.* 33 1–12.

[B84] Okoye N. J. C. Okoye M. O. (2018). *Sustainability of Impact Factor Metrics in Appraisals, Tenure and Promotions of Academic Librarians in South East Nigerian Federal Universities.* Nsukka: University of Nigeria.

[B85] Orji M. G. Yakubu G. N. (2020). Effective stress management and employee productivity in the nigerian public institutions; a study of national galary of arts, Abuja, Nigeria. *Budapest Int. Res. Critics Inst.* 3 1303–1315. 10.33258/birci.v3i2.975

[B86] Orkibi H. Brandt Y. I. (2015). How positivity links with job satisfaction: preliminary findings on the mediating role of work-life balance. *Eur. J. Psychol.* 11 406–418. 10.5964/ejop.v11i3.869 27247666 PMC4873052

[B87] Rahimi E. Bavaqar S. (2010). Effects of yoga on anxiety and depression in women. *Br. J. Sports Med.* 44 (Suppl. 1), i68–i69. 10.1136/bjsm.2010.078725.227

[B88] Rao J. V. Chandraiah K. (2012). Occupational stress, mental health and coping among information technology professionals. *Indian J. Occup. Environ. Med.* 16 22–26. 10.4103/0019-5278.99686 23112503 PMC3482704

[B89] Ross A. Thomas S. (2010). The health benefits of yoga and exercise: a review of comparison studies. *J. Altern. Complement. Med.* 16 3–12. 10.1089/acm.2009.0044 20105062

[B90] Ruotsalainen J. H. Verbeek J. H. Marine A. Serra C. (2015). Preventing occupational stress in healthcare workers. *Cochrane Database Syst. Rev.* 11:CD002892. 10.1002/14651858.CD002892.pub5 17054155

[B91] Sharma V. Shrivastava S. MalhSharma U. K. Shrivastava S. Malhotra S. Singh R. (2010). Yoga and cognitive behaviour techniques for academic stress and mental wellbeing among school students. *Delhi Psychiatry J.* 13 12–19.

[B92] Shivangana V. (2018). *Yoga for beginners: 10 basic poses (Asanas) to get you started.* Available online at: https://food.ndtv.com/health/yoga-for-beginners-10-basic-poses-to-get-you-started-1229662 (accessed October 4, 2020).

[B93] Slišković A. Seršić D. (2011). Work stress among university teachers: gender and position differences. *Arch. Industr. Hygiene Toxicol.* 62 299–307. 10.2478/10004-1254-62-2011-2135 22202463

[B94] Stults-Kolehmainen M. A. Sinha R. (2014). The effects of stress on physical activity and exercise. *Sports Med.* 44 81–121. 10.1007/s40279-013-0090-5 24030837 PMC3894304

[B95] Wanjiku M. G. (2015). *Institutional Factors Influencing Academic Governor’s Occupational Stress in Secondary Schools in Dagoreti Subcounty Kenya.* Dissertation. Nairobi: University of Nairobi.

[B96] Winter R. (2009). Academic manager or managed academic? Academic identity schisms in higher education. *J. Higher Educ. Policy Manag.* 31 121–131. 10.1080/13600800902825835

[B97] World Health Organization [WHO] (2016). *Depression.* Available online at: http://www.who.int/mediacentre/factsheets/fs369/en/. (accessed July 20, 2018).

[B98] Wu Z. Sullivan S. G. (2009). “Community-based intervention trials in low-and middle-income countries,” in *Oxford Textbook of Public health, Volume 2: The Methods of Public Health*, Ed. 5 Edn, eds Holland W. W. Detels R. (New York, NY: Oxford University Press), 567–579. 10.1093/med/9780199218707.003.0035

